# Enhancing Arthropod Diversity and Sorghum Quality in Northern Jiangsu, China: The Benefits of Green Pest Management Revealed Through Metabarcoding

**DOI:** 10.3390/ijms26072977

**Published:** 2025-03-25

**Authors:** Qian Jin, Yuxuan Zheng, Mingquan Pan, Xiaoman Zhang, Aibing Zhang, Shangkun Lai

**Affiliations:** 1 Suqian Institute of Agricultural Sciences, Jiangsu Academy of Agricultural Sciences, Suqian 223800, China; jinhongyu2001@163.com (Q.J.); pmq207@126.com (M.P.); 2 College of Life Sciences, Capital Normal University, Beijing 100048, China; zhengyx1612@163.com; 3 Hebei Key Laboratory of Animal Physiology, Biochemistry and Molecular Biology, College of Life Sciences, Hebei Normal University, Shijiazhuang 050024, China; zhangxiaoman@hebtu.edu.cn

**Keywords:** arthropod diversity, DNA metabarcoding, green pest management, sorghum, species diversity

## Abstract

Sorghum is a key global crop with substantial economic importance. Implementing green pest management for sorghum is crucial for promoting ecological balance and reducing reliance on chemical pesticides. This study assesses the impact of green pest management on arthropod biodiversity and sorghum yield and quality. Over two years, using Malaise trapping and DNA metabarcoding, we found that green pest management significantly enhanced arthropod diversity, increasing species richness by 5.63% and shifting species composition, notably increasing the abundance of Hymenoptera. Although sorghum yield metrics were higher in the green group compared to the chemical control group, these differences were not statistically significant. However, the green group exhibited improved quality with lower crude fat (3.63% vs. 4.08% in the chemical control group) and higher levels of crude protein (9.18% vs. 9.13%), starch (73.69% vs. 73.41%), and amylopectin (98.53% vs. 98.34%). These findings underscore the benefits of green pest management in fostering biodiversity and enhancing sorghum quality. Future research should focus on optimizing biodiversity-driven agroecosystem resilience and scaling these strategies across diverse agricultural systems.

## 1. Introduction

Sorghum, a critical crop with major production centers in the United States, Canada, Australia, and China, holds significant economic and nutritional value [1,2,3]. It serves as a staple food and a vital source of livestock feed, particularly in arid and semi-arid regions. In addition, sorghum is an important raw material for brewing [4,5], with cultivation in China predominantly concentrated in the north and southwest regions. Despite its resilience, sorghum cultivation faces considerable challenges from pests, especially lepidopteran insects such as the *Ostrinia furnacalis* (Asian corn borer), *Synanthedon exitiosa* (peach borer), and *Spodoptera frugiperda* (armyworm) [3,4,6]. These pests threaten crop yields by feeding on leaves and boring into stalks and panicles.

The impact of these pests highlights the urgent need for effective pest management strategies. Traditional pest control methods often rely on chemical pesticides, which can harm the environment and impact non-target species [7], including beneficial arthropods. Because of sorghum’s sensitivity to various pesticides, such as pyrethroids, organophosphates, carbamates, and Bordeaux mixture, including lime sulfur, compounds are a significant issue [3]. Furthermore, modern pesticide formulations, often containing multiple active ingredients, similarly cause phytotoxicity if used improperly. Additionally, the common practice of applying pesticides through stem and leaf spraying is often ineffective for sorghum varieties with compact panicles that conceal pests, meaning that only a small portion of the pesticide reaches the hidden pests within the panicles. This leads to inefficient pesticide utilization, increased frequency of applications, and the potential for pest resistance development [5,8]. This results in non-point source pollution, excessive pesticide residues, and higher production costs, ultimately reducing sorghum farming profitability.

To mitigate these negative impacts, green pest control methods emerged as a promising alternative [9]. These sustainable approaches emphasize the use of biological control agents and environmentally friendly practices to manage pest populations while preserving ecological balance [10,11]. Unlike traditional methods that rely heavily on chemical interventions, green pest control methods aim to reduce environmental harm and minimize the use of synthetic pesticides. Changes in arthropod biodiversity are increasingly recognized as key indicators of agricultural sustainability, offering valuable insights into the health and stability of agroecosystems [12,13]. By monitoring these shifts, we can better understand the impact of pest control strategies on the broader agroecosystem.

DNA metabarcoding emerged as a powerful tool for evaluating insect diversity, providing a comprehensive and efficient method for identifying and quantifying a wide range of arthropod species [14,15,16,17]. It works by designing target primers and focusing on specific regions of the insect genome, capturing and decoding key genetic information [18]. This allows precise identification of species within complex biological samples, offering a detailed view of the arthropod communities present in various environments. This technology enhances our ability to monitor biodiversity and assess the impacts of agricultural practices on arthropod communities, thereby supporting the development of more sustainable pest management strategies.

Here, we employed Malaise trapping [19] and DNA metabarcoding [16,17] techniques to analyze arthropod communities in sorghum fields over two years. We determined the composition of arthropods in these fields and assessed the impact of green pest control methods on the diversity of insect orders such as Lepidoptera and Hymenoptera. We also evaluated the effects of green pest control on sorghum yields, underscoring the importance of integrating sustainable pest management practices to enhance crop health and farming efficiency.

## 2. Results

### 2.1. Arthropods Dominate Across Different Years

**Figure 1:**
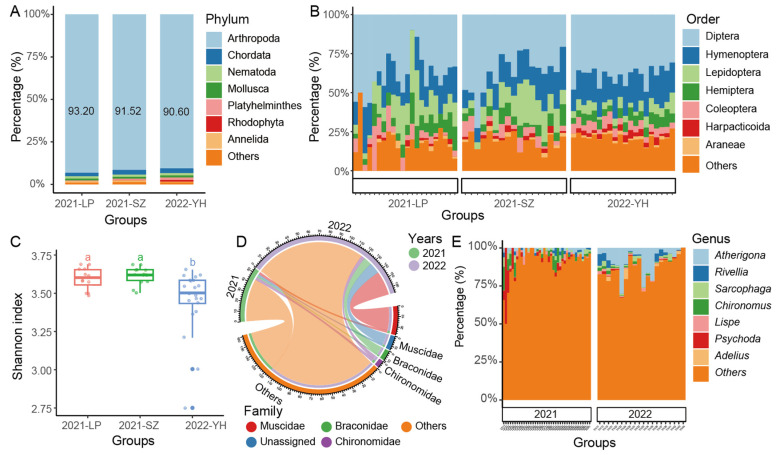
Species composition and diversity comparison between different years (2021 and 2022) and fields (LP, SZ, and YH). Arthropods, which made up over 90% of the collected samples, highlighting the differences across fields and years. (**A**) Phylum composition across different years and fields, Different letters indicate statistically significant differences at *p* < 0.05. (**B**) Order composition at the order level across different years and fields. (**C**) Alpha diversity (Shannon index) differences across different years and fields. (**D**) Family-level composition across both years, showing the abundance of key arthropod families. (**E**) Genus-level composition for 2021 and 2022, focusing on the most abundant genera and their variation between years.

To investigate the diversity of arthropods in sorghum fields over different years, we conducted a Malaise traps survey in two fields (LP and SZ) in 2021 and in one field (YH) in 2022. Our results indicate that the Malaise traps were highly effective in capturing arthropod species, with arthropods comprising more than 90% of the collected samples (Figure 1A). This high efficiency might indicate the dominance of arthropods in the sorghum field ecosystem and the seasonal climatic conditions (Table 1). Among the collected samples, the most dominant orders were Diptera, Hymenoptera, and Lepidoptera, leading us to focus subsequent analyses on these key arthropod taxa (Figure 1B).

In comparing diversity across the different fields and years, we observed no significant differences in the Shannon diversity index between the different fields (LP, SZ, and YH) surveyed in 2021 (Figure 1C). This suggests that, within the same year, the arthropod communities in different locations were relatively consistent in terms of species diversity. A clear distinction emerged when comparing the two years: the Shannon diversity index for 2021 was significantly higher than that for 2022, indicating that arthropod diversity was notably richer in 2021. However, it is important to note that the actual difference in the Shannon index was relatively small, suggesting that the effect size, although statistically significant, was low.

Further analysis revealed that the most abundant arthropod families across both years were Muscidae (house flies), Braconidae (parasitic wasps), and Chironomidae (non-biting midges). These families were consistently present in large numbers, contributing substantially to the overall community structure (Figure 1D). On a more detailed level, we identified several genera that were particularly dominant in both years, including *Autherigona*, *Rivellia*, *Sarcophaga*, *Chironomus*, *Lispe*, *Psychoda*, and *Adelius* (Figure 1E), though their relative abundance varied between 2021 and 2022. For instance, *Psychoda* and *Chironomus* were notably more prevalent in the LP field samples from 2021, indicating a localized surge of these genera that year. In contrast, *Autherigona* showed a pronounced dominance in 2022, appearing in 20 out of the 21 samples from that year, suggesting a shift in community structure between the two years. This may be suggested a significant shift in the arthropod community structure between 2021 and 2022, with *Autherigona* becoming a dominant species in 2022 compared to 2021.

### 2.2. Increased Arthropod Diversity and Hymenoptera Dominance in Green Groups

**Figure 2:**
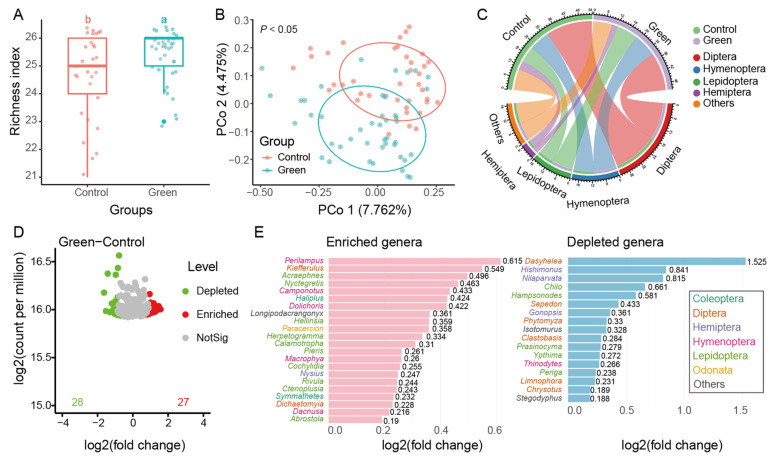
Species comparison between green and chemical control management. Compared to the chemical control group, the green management groups showed an enrichment of more Hymenopte ra insects. (**A**) Richness index. Different letters indicate statistically significant differences at *p* < 0.05. (**B**) Beta diversity. (**C**) Species composition at the order level (**D**) volcano plot showing OTU differences; and (**E**) compared to control, the depleted and enriched genera in green groups. *p* value was calculated by Kaiser–Meyer–Olkin test.

We compared the alpha and beta diversity across different fields (LP, SZ, LX, and PW) in 2021, focusing on chemical control groups (LP, SZ) and green groups (LX, PW). No significant differences were observed in the richness index (Figure S1A) or beta diversity (Figure S1B) within either the control (LP, SZ) or green (LX, PW) groups, indicating that our sampling was representative and that both groups exhibited reproducibility. We then analyzed the differences between the green groups (LX, PW) and the chemical control groups (LP, SZ). The green groups had a significantly higher richness index compared to the chemical control groups (Figure 2A, 25.31 vs. 23.96), and their beta diversity was significantly distinct from the chemical control groups (*p* < 0.05, Figure 2B), although the explanation of the differences was limited. Diptera, Hymenoptera, Lepidoptera, and Hemiptera were the dominant order groups in both the chemical control and green groups (Figure 2C).

To highlight the specific differences between the chemical control and green groups, we identified 28 OTUs that were depleted and 27 OTUs that were enriched in the green group compared to the chemical control group (Figure 2C). To further examine these OTUs, we removed species classified only at the genus level and combined taxa within the same genus. This revealed 22 enriched genera and 17 depleted genera (Figure 2D,E) in the green group. *Perilampus* (Hymenoptera, Perilampidae) was the most enriched genus in green group, while *Dasyhelea* (Diptera, Ceratopogonidae) was the most enriched genus in the chemical control group. Among these genera, those in the order of Hymenoptera were predominantly enriched in the green group, while genera in Diptera and Lepidoptera were more abundant in the chemical control group, showing higher log2 fold changes.

Additionally, there were no significant differences in the richness index among different months across July, August, September, and October (Figure S1C). However, beta diversity was primarily influenced by the month, with July showing significant differences compared to other months (Figure S1D). We further compared differences between groups across different times (July, August, to October). Although no differences in the richness index were observed between groups at different times, the green group had higher values from August to October compared to the chemical control group (Figure S1E). Significant differences in beta diversity were observed between groups and across different times, with beta diversity values being more similar along the PCo1 axis at different times (Figure S1F), suggesting that community composition at these times was more alike based on the first principal coordinate. Notably, from August to October, the green group had a higher percentage of Hymenoptera species compared to other periods.

### 2.3. Green Groups Show Higher Sorghum Yield and Improved Grain Quality Trends

**Figure 3:**
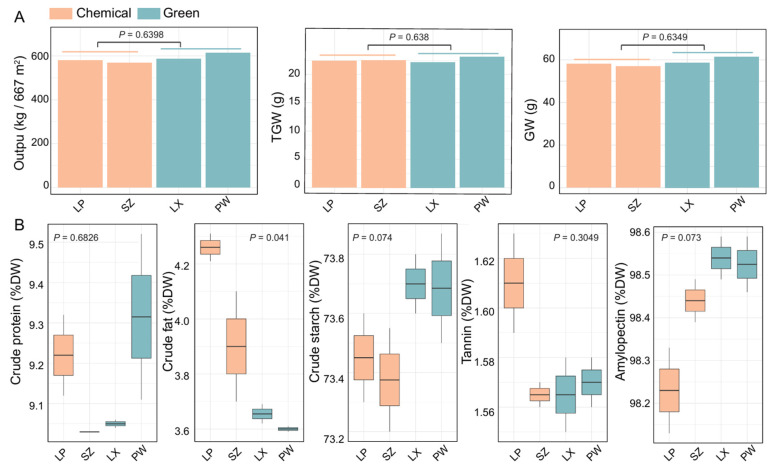
Comparison of sorghum yield and quality between green and chemical control groups. Sorghum yield and quality in the green management places are higher than those in the chemical control group. (**A**) Comparison of sorghum yield, including output (kg/667 m^2^), thousand-grain weight (TGW), and grain weight (g). (**B**) comparison of sorghum quality, including crude protein content (% DW), crude fat content (% DW), crude starch content (% DW), tannin content (% DW), and amylopectin proportion of total starch (%). DW, dry weight. *p* < 0.001 by *t*-test (two-sided).

We then compared sorghum yield and quality between the green and chemical control groups using data from four locations (chemical control group: LP, SZ; green group: LX, PW) for the years 2021–2022. To evaluate sorghum yield (Figure 3A), we analyzed output (kg/667 m^2^), 1000-grain weight (g), and grain weight (g). Although no significant differences were observed between the groups, the green group showed higher average values, with output (557.30 kg/667 m^2^), 1000-grain weight (22.45 g), and grain weight (55.73 g) compared to the chemical control group’s average values of output (541.45 kg/667 m^2^), 1000-grain weight (22.30 g), and grain weight (54.15 g).

We also compared sorghum quality between the green and chemical control groups using the following metrics: crude protein content (% DW), crude fat content (% DW), crude starch content (% DW), tannin content (% DW), and amylopectin proportion of total starch (% DW). Here, DW stands for dry weight (Figure 3B). Our analysis revealed that the green group had a significantly lower crude fat content compared to the chemical control group (*p* = 0.041). Although no other metrics showed significant differences, the green group had higher average values for crude protein content (% DW), crude starch content (% DW), and amylopectin proportion of total starch (% DW), while the tannin content (% DW) was lower compared to the chemical control group (Figure 3B). In summary, the green group showed improved quality with lower crude fat (3.63 vs. 4.08 in the control group) and higher levels of crude protein (9.18 vs. 9.13%), starch (73.69 vs. 73.41), and amylopectin (98.53 vs. 98.34).

## 3. Discussion

Our analysis demonstrates that differences in arthropod diversity between years were more pronounced than differences between fields within the same year. However, we acknowledge that the limited number of sampling sites, traps, and the unbalanced sampling effort between years may influence the robustness and generalizability of our findings. To address these limitations, we clarified that our results should be interpreted cautiously and emphasized the need for further research with expanded and balanced sampling designs to validate the observed trends.

The significant variation in arthropod diversity between 2021 and 2022, despite similar diversity within fields in a given year, highlights the influence of temporal factors on arthropod communities. In 2021, there was a distinct increase in the abundance of certain genera, such as *Psychoda* and *Chironomus*, particularly in the LP field. However, in 2022, *Autherigona* emerged as the dominant genus, with a substantial presence in the majority of samples. This variation underscores the importance of year-to-year fluctuations in species composition and community structure, which may be influenced by environmental changes or differences in agricultural management practices.

Our comparison between green and chemical control groups revealed that green pest management practices lead to increased arthropod richness and a higher dominance of Hymenoptera. This plays a crucial role in natural pest control and contribute significantly to ecosystem health [11,20,21]. The increased presence of Hymenoptera in green management fields suggests that these practices enhance ecosystem service provision, particularly through natural pest control mechanisms. Nowadays, biological control through natural enemies, such as the use of parasitoids and predators, has become a key component of sustainable crop production systems [22,23]. At our sampling sites, we also implemented the release of *Trichogramma*, a well-known biological control agent, to further support pest management (see Methods and Materials). The positive impact of green pest management on the abundance of Hymenoptera underscores the effectiveness of integrating natural enemies into pest control strategies.

Our findings indicate that green pest management not only promotes increased arthropod diversity, particularly with a rise in Hymenoptera, but also has potential impacts on sorghum quality. While no significant differences in sorghum yield were observed between green and chemical control groups, the green group consistently exhibited higher average values in key quality metrics, including crude protein, crude starch, and amylopectin proportions, alongside a reduction in crude fat content. This raises the question of whether the shift in arthropod community structure, driven by green pest management, is influencing crop quality [24].

One possible mechanism behind these changes could be the increased abundance of beneficial Hymenoptera, such as parasitoids and pollinators, which enhance natural pest control and contribute to plant health [20,21,25]. By reducing pest pressure, these insects may improve nutrient allocation in plants, affecting crop traits such as protein and starch content. For instance, research demonstrated that increased plant diversity may have positive effects on crop yield, while arthropod herbivory may negatively impact yield [24,26]. Additionally, research into plant–insect interactions suggests that insect herbivory can alter plant biochemical pathways, influencing nutrient composition. Green pest management, by fostering a balanced arthropod community, may mitigate these negative effects of herbivory.

Although the exact mechanisms are not fully understood, promoting beneficial arthropods through sustainable pest control strategies likely enhances ecological processes that support plant health, contributing to the observed improvements in crop quality [11,23]. Our study supports the idea that biodiversity, driven by practices such as green pest management, plays a crucial role in improving both ecosystem services and crop performance. This aligns with broader research advocating for sustainable agriculture practices to enhance long-term yield and quality.

Additionally, future studies should aim to include a greater number of sites and replicates within each treatment type to disentangle treatment effects from site-specific environmental factors. This would help address the limitations of spatial and environmental variability, ensuring a more robust and comprehensive understanding of how pest management strategies influence arthropod communities and agricultural outcomes. Expanding sampling efforts across diverse geographic regions and temporal scales will also provide greater insight into the consistency and generalizability of these findings.

## 4. Materials and Methods

### 4.1. Basic Information of Sampling Places

Samples for this study were collected from the liquor-making manufacturing base at the Yanghe Agricultural High-tech Zone (18 m above sea level, 118.432° E, 33.783° N) and Siyang County in Suqian City, Jiangsu Province (17 m above sea level, 118.32° E, 33.97° N). These sites are located in the Huaihe Plain, characterized by a warm temperate monsoon climate with an average annual temperature of 14.2 °C, average annual precipitation of 910 mm, total annual sunshine hours of 2291 h, and a crop growth period of 310.5 days per year. Sorghum, a local specialty crop, is mainly used for making Baijiu (Chinese liquor). Two green pest control areas (LX, PW) and non-green pest control areas, also called the chemical control group (YH, LP, and SZ) were set up in both bases (Table S1, Figure S2).

### 4.2. Green and Non-Green Pest Control

#### 4.2.1. Green Pest Control Methods

**Figure 4:**
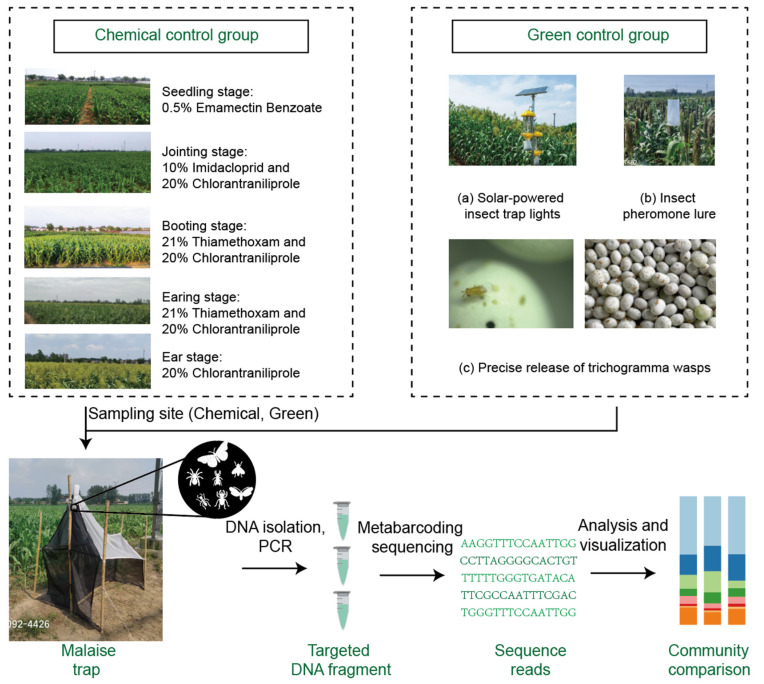
Sampling methods.

In green-controlled fields, no chemical control methods are used. Instead, the following green pest control techniques are employed. Green pest management refers to pest control practices that minimize the use of chemical pesticides and prioritize environmentally sustainable methods. This approach focuses on ecological balance, using non-toxic strategies to reduce pest populations and promote long-term sustainability. It integrates methods such as biological control, physical barriers, and the use of natural pest deterrents. (a) Solar-powered insect trap lights. This method takes advantage of the tendency of Lepidopteran pests to be attracted to light. One solar-powered insect trap light, integrating wind and solar power, was installed for every 50 acres of land. These lights are purchased from Nanjing Liye Pest Control Co., Ltd., Nanjing, China. (b) Insect pheromone lure technology. For each pest species being controlled (specifically corn borers (*Ostrinia furnacalis*) and peach borers (*Conogethes punctiferalis*)), 2–3 traps are installed per acre. Each trap is equipped with a species-specific pheromone lure to attract the targeted pest. The lure cartridges are replaced every 3–5 weeks. Both the traps and the pheromone lures are sourced from Nanjing Liye Pest Control Co., Ltd., Nanjing, Jiangsu province, China. (c) Precise release of trichogramma wasps. *Trichogramma* wasps (specifically *Trichogramma chilonis* and *Trichogramma dendrolimi*, *Trichogramma dendrolimi* carrying *Beauveria bassiana*) were released four times in the green-controlled sorghum fields between July and October of 2021. The release dates were 25 June, 1 July, 8 July, and 16 August. Each hectare received approximately 225,000 wasps (equivalent to 15,000 wasps per acre) each time. Five release points were set up per acre (Figure 4).

#### 4.2.2. Non-Green Pest Control Methods

In fields without green control, chemical pesticides were applied throughout the growing season with five sprayings targeting key growth stages: at the seedling stage, 0.5% emamectin benzoate was used; at the jointing stage, 10% imidacloprid and 20% chlorantraniliprole were applied; at the booting stage, 21% thiamethoxam and 20% chlorantraniliprole were used; at the earing stage, 21% thiamethoxam and 20% chlorantraniliprole were applied; and at the ear stage, 20% chlorantraniliprole was used. The dosages ranged from 120 to 450 mL/ha depending on the growth stage, ensuring targeted pest management (Figure 4, Table 2). This group was therefore referred to as the chemical control group.

### 4.3. Collection of Arthropods

#### 4.3.1. Sampling and Field Experiment Design

Arthropod samples were collected using Malaise traps. Three traps were randomly set up at each sampling site, positioned 10 m from the field edge to minimize edge effects. Each Malaise trap was 0.9–1.8 m in height and 1–1.5 m in width, with a bottle at the top of the trap, containing ethanol to preserve the captured arthropods. The samples were collected every two weeks, resulting in a total of 104 Malaise trap bottles (Table S1). Specifically, 83 bottles were collected from four sites sampled in 2021 (LP; SZ; LX; PW) between 1 July and 28 October. Additionally, 21 bottles were collected from one site (YH, YangHe) sampled in 2022 between July 15 and October 25. The site sampled alone in 2022 was chosen for comparing pest species composition across different years or evaluating a treatment effect at a particular location.

This study was conducted at two sorghum cultivation bases in Suqian City, Jiangsu Province: The Yanghe Agricultural High-tech Zone Winemaking Raw Grain Base (altitude 18 m, 118.432° E, 33.783° N) and the Siyang County South Base (altitude 17 m, 118.32° E, 33.97° N). Both bases included green control areas (LX, PW) and chemical control areas (YH, LP, and SZ; specific treatments detailed in Section 2.2 “Green vs. Chemical Control”). Each treatment zone comprised three parallel trials, totaling 15 experimental plots (Figure S2). The sorghum cultivar Qianniangliang 1, a glutinous variety bred jointly by the Suqian Institute of Agricultural Sciences (Jiangsu Academy of Agricultural Sciences) and Jiangsu Yanghe Distillery Co., Ltd., was uniformly planted. Experimental fields were leveled with homogeneous soil fertility. All agronomic practices (except pest control) were consistent across green and chemical control plots. Prior to sowing, 40–50 kg of 45% compound fertilizer per 667.7 m^2^ was applied as base fertilizer during soil rotary tillage. Sowing occurred from June 15 to 17 with row spacing of 45–50 cm, plant spacing of 13–15 cm, and sowing depth of 3–5 cm. Comprehensive soil physicochemical profiles for each experimental site are detailed in Table S2.

#### 4.3.2. DNA Extraction, PCR Amplification, High-Throughput Sequencing

Each sample collected from the Malaise traps was soaked in 500 mL of sterile water at 4 °C for 24 h to remove ethanol. This was repeated three times, such that residual ethanol was removed. The total DNA in the lysate, obtained by lysing the insect bodies with 10 mL of lysis buffer GA (50 mM Tris-HCl (pH 8.0), 100 mM NaCl, 10 mM EDTA, 1% SDS, and 5% Polyvinylpyrrolidone (PVP)), was extracted using the membrane adsorption method. PCR amplification was performed using the primers LCO1490 (GGTCA ACAAA TCATAA AGATA TTGG) and HCO2198 (TAAAC TTCAG GGTGA CCAAA AAATCA) [27]. The PCR reaction system was prepared in a total volume of 50 μL, which included 2 μL of DNA template, 25 μL of 2 × EX Taq PCR MasterMix, and 1 μL each of forward and reverse primers (10 μmol/L). The volume was then adjusted with double-distilled water to reach the final 50 μL. The PCR amplification conditions were as follows: an initial denaturation at 95 °C for 5 min, followed by 40 cycles of denaturation at 95 °C for 30 s, annealing at 50 °C for 30 s, and extension at 72 °C for 1 min. After the 40 cycles, a final extension step was performed at 72 °C for 10 min. The PCR products were then purified, and the purified amplicons were sequenced using the Illumina MiSeq PE300 platform (Illumina, Inc., San Diego, CA, USA) for high-throughput sequencing.

#### 4.3.3. Metagenomic Data Processing

Raw sequencing data were processed using several bioinformatics tools to ensure high-quality results. First, raw sequences were quality-controlled using Trimmomatic v0.39 software [28], where reads were trimmed based on base quality scores and adapter sequences were removed. Data quality before and after trimming was assessed using FastQC v0.11.5 [29], which provides a detailed report on the overall quality of the sequence data, including read length distribution, GC content, and sequence duplication levels. To further refine the data, the FASTX-Toolkit v1.4.1 was used for adapter removal, which eliminates residual adapter sequences that may interfere with downstream analysis. For paired-end sequence splicing, we employed FLASH v2.2.0 [30], achieving a splicing efficiency greater than 95%, allowing for the reconstruction of longer, contiguous sequences from paired-end reads.

To remove potential chimeric sequences—artifacts formed during PCR amplification—we utilized VSEARCH v2.7.1 [31]. This step ensures that the data used in downstream analyses are free of spurious sequences that could lead to inaccurate taxonomic classification. After these processing steps, we obtained 8488 sequences, including 4723 from 2021 and 3765 from 2022. All raw sequences are available online on NCBI databases (https://submit.ncbi.nlm.nih.gov/subs/sra/SUB14716726/overview (accessed on 17 March 2025)). The associated numbers are from SRR30663504 to SRR30663512.

#### 4.3.4. BOLD System MOTUs Division

All sequences were submitted to the BOLD system [32] for species division via the website http://www.boldsystems.org (accessed on 13 December 2024). After chimera detection, the remaining high-quality sequences were clustered into operational taxonomic units (OTUs) at 97% sequence identity [33]. The bold.py program was utilized, with input data in ‘.fas’ format files (for example, XX.fas as input file, with filenames inside the file such as ‘>22YH030930.249′), producing four output files: (a) XXouts.fa; (b) XXBOLD.sum.fas; (c) Xxouts.fa.error.fas; and (d) XXBOLD.fas, where ‘XXBOLD.fas’ represents the final BOLD results, with specific filenames containing six-level annotation results, such as ‘>22YH010715.1_Arthropoda_Insecta_Hymenoptera_Formicidae_Rhytidoponera_XX(55.56)’.

#### 4.3.5. Downstream Analysis of Metagenomic Data

Downstream analysis of community composition was performed using the Phyloseq package V. 1.44.0 [34]. Specifically, an OTU table was generated by clustering OTUs at 97% similarity to document the relative abundance of each OTU in each sample [35], along with the taxonomy of the OTUs. After taxonomic identification, differentially abundant OTUs were selected as key OTUs for further analysis. Our analyses included species composition, diversity assessment, inter-group comparisons, heatmap visualization, and identification of key OTUs. Briefly, for alpha diversity, we assessed differences in the Shannon [36] and richness indices [37]. Beta diversity was analyzed using principal coordinates analysis (PCoA) based on weighted UniFrac distances, with statistical significance evaluated through analysis of similarity (ANOSIM), to explore differences in community composition between treatment groups and year [38]. Statistical analyses were performed using R (v4.3.1), and relevant packages, such as *Microbiome*, *amplicon*, *microeco*, and *vegan* packages were employed for taxonomic and diversity analyses, and visualizations were created using *ggplot2*, *dplyr*, *RColorBrewer*, and *ggpubr*, ensuring clear and publication-quality graphical outputs [39,40].

### 4.4. Sorghum Yield and Quality Calculations

#### 4.4.1. Sample Collection

The grain is harvested when it reaches full maturity. After harvesting, it is promptly dried, threshed, and weighed. The yield is calculated at a standard 14% moisture content and expressed in kilograms per hectare, rounded to one decimal place. During harvest, the outermost rows on both sides (one row on each side) are removed, and only the central rows are used for yield calculation.

#### 4.4.2. Data Analysis

We calculated the yield, thousand grain weight (TGW), grain weight, and grain quality. For yield, each harvest covered an area of no less than 1200 square meters. At each sampling point, three plots were randomly selected, and the actual yield from these plots was measured and converted to yield per hectare (1 hectare = 10,000 square meters). The TGW of fully mature grains was measured when the grain moisture content reached 14% and expressed in grams (g). The weight of the grain from a single ear was measured in grams. From each plot, a 1 kg sample of grain is collected and sent to the Grain and Products Quality Supervision, Inspection, and Testing Center of the Ministry of Agriculture and Rural Affairs (Harbin) for analysis. The following quality indicators were tested: crude protein (dry basis), crude fat (dry basis), crude starch (dry basis), amylopectin (as a percentage of starch), and tannin (dry basis). Differences in sorghum yield and productivity between green and non-green control fields were analyzed using the Wilcoxon rank sum test. Specifically, for yield, TGW, and grain weight, we used the Wilcoxon rank sum test to compare the differences between the two treatment groups (green vs. non-green control). For the grain quality parameters (crude protein, crude fat, crude starch, amylopectin, and tannin), a similar Wilcoxon rank sum test was applied to assess the statistical significance of the differences between the groups. All tests were conducted at a significance level of *p* = 0.05.

## 5. Conclusions

In conclusion, our study highlights the significant potential of green pest management for improving arthropod diversity and sorghum quality. While no statistically significant differences were observed in sorghum yield between green and chemical control groups, the green pest management approach consistently enhanced key quality traits, including crude protein, starch, and amylopectin content, while reducing crude fat. The increased abundance of beneficial Hymenoptera suggests that green pest management supports natural pest control and ecosystem health. These findings provide valuable insights into the ecological benefits of green pest management, advocating for its integration into sustainable agricultural practices. Future research should focus on optimizing these methods and understanding the mechanisms behind their positive impact on crop health and yield.

## Figures and Tables

**Table 1:** Monthly Variations in Accumulated Rainfall (mm) and Average Temperature (°C) During the Experimental Season at Yanghe Yuangliang and Siyang Chengnan Bases.

Year	Field	Month	Accumulated Rainfall (mm)	Average Maximum Temperature (°C)	Average Minimum Temperature (°C)	Average Temperature (°C)
2021	Yanghe Agricultural High-tech Zone (LP + LX)	Jul.	612.4	32.2	24.5	27.5
		Aug.	111.9	31.7	23.7	26.9
		Sep.	178.7	30.2	21.6	24.8
		Oct.	81.8	23.6	13.8	17.6
2021	Siyang County in Suqian City (SZ + PW)	Jul.	867.6	31.4	24.6	27.5
		Aug.	117.5	30.7	23.6	26.8
		Sep.	203.4	29.2	21.3	24.6
		Oct.	97.5	22.7	13.3	17.3
2022	Yanghe Agricultural High-tech Zone (YH)	Jul.	287.9	33	25.2	28.5
		Aug.	90.8	33.4	25.6	29
		Sep.	3.9	27.8	18.9	22.9
		Oct.	49.4	22.2	11.9	16.3

**Table 2:** The application frequency, time, pesticide and dosage of normal chemical control.

Frequency	Time	Pesticide	Dosage of Pesticide
First time	Seedling stage	0.5% Emamectin benzoate	450 mL/ha
Second time	Jointing stage	10% Imidacloprid, 20% Chlorantraniliprole	225 g/h, 120 mL/ha
Third time	Booting stage	21% Thiamethoxam, 20% Chlorantraniliprole	120 mL/ha each
Fourth time	Earing stage	21% Thiamethoxam, 20% Chlorantraniliprole	120 mL/ha, 150 mL/ha
Fifth time	Ear stage	20% Chlorantraniliprole	150 mL/ha

mL, milliliters; g, gram; and ha, hectare.

## Data Availability

All data and drawing code are provided in the paper and Supplementary Materials. The raw sequence data project had been deposited at GenBank under BioProject accession number PRJNA1156354, and the SRR numbers were from SRR30663504 to SRR30663512.

## Supplementary Materials

The following supporting information can be downloaded at: https://www.mdpi.com/article/10.3390/ijms26072977/s1.
