# Supplementary material for: Enhancing Arthropod Diversity and Sorghum Quality in Northern Jiangsu, China: The Benefits of Green Pest Management Revealed Through Metabarcoding
Source: Int J Mol Sci. 2025 Mar 25;26(7):2977. doi: 10.3390/ijms26072977 (PMC11988586; doi:10.3390/ijms26072977)
Supplement: Supplementary file 1 [file ijms-26-02977-s001.zip › ijms-3399673-supplementary.pdf]

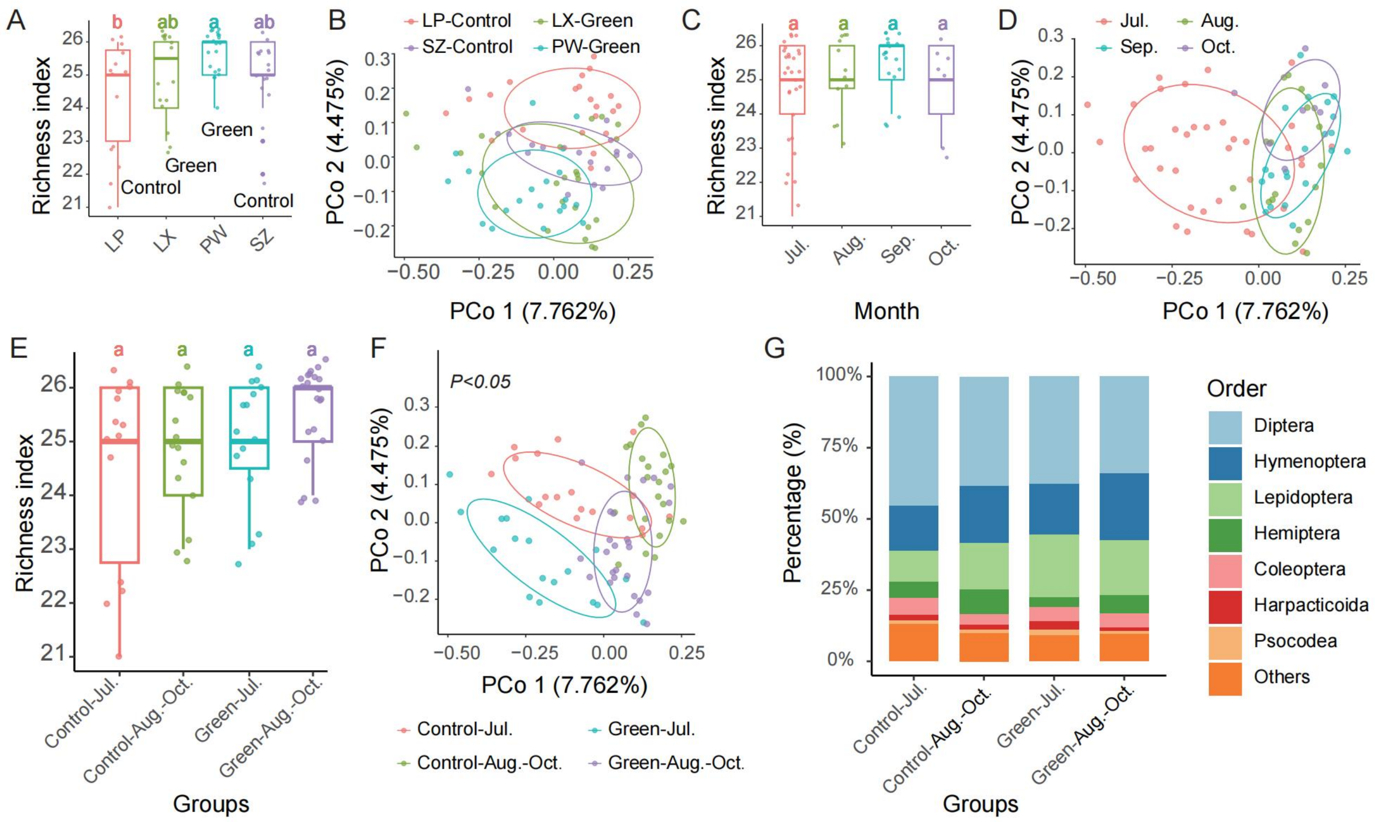

Figure S1. Comparison of species diversity between control and green management across various locations and months. A. Richness index across different locations; B. Beta diversity across different locations; C. Richness index across different months; D. Beta diversity across different months; E. Comparison of the richness index between control and green groups across different months; F. Comparison of beta diversity between control and green groups across different months; G. Species composition across different months for control and green groups.

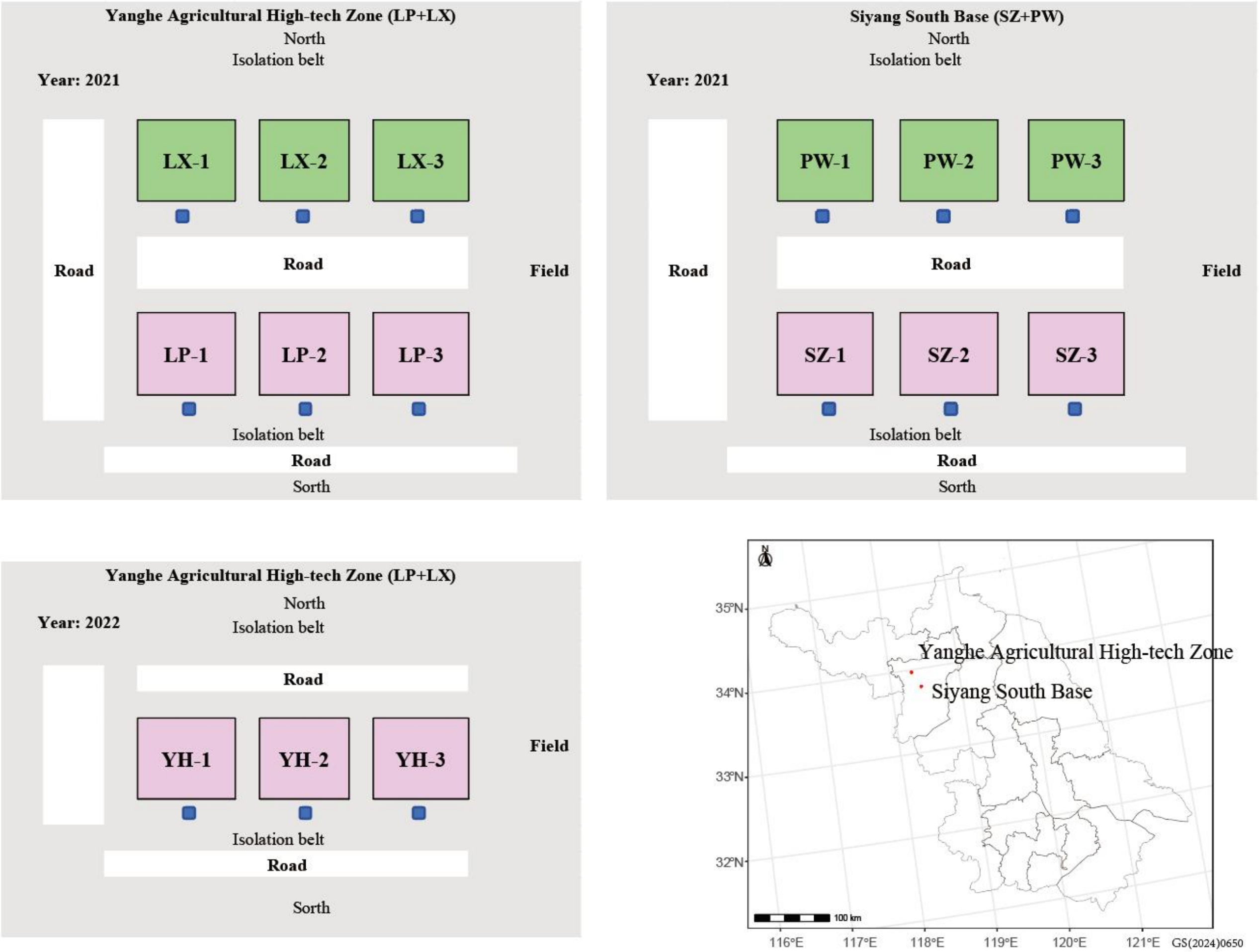

Figure S2. The basic information of sampling sites.

Table S1. Sampling collection information.

Field	Full name	Group	Year	Month	Samples	Summary samples
YH	YangHe	Control	2022	Jul.	3	21
				Aug.	6	
				Sep.	6	
				Oct.	6	
LP	LiuLiPeng	Control	2021	Jul.	6	22
				Aug.	6	
				Sep.	6	
				Oct.	4	
SZ	SanZhuang	Control	2021	Jul.	6	17
				Aug.	6	
				Sep.	3	
				Oct.	2	
LX	LiuLiPeng Xi	Green	2021	Jul.	6	24
				Aug.	6	
				Sep.	6	
				Oct.	6	
PW	PeiWei	Green	2021	Jul.	6	20
				Aug.	6	
				Sep.	4	
				Oct.	4	

TableS2 Comprehensive soil physicochemical profiles for each experimental site.

Locus	pH	Organic （g/kg）	Total Nitrogen （mg/kg）	Total Phosphorus （%）	Total Potassium （%）	Available Nitrogen （mg/kg）	Available Phosphorus （mg/kg）	Available Potassium （mg/kg）
YH	7.94	17.7	1060	0.052	1.89	92.3	5.9	112
LP	7.85	19.1	1058	0.039	1.98	92.6	6.9	133
SZ	7.72	14.4	805	0.070	1.72	69.3	19.9	159
LX	7.75	19.1	1130	0.053	2.05	93.7	6.0	103
PW	7.78	15.2	836	0.079	1.78	64.9	21.1	124
